# Whole-Genome Sequence and Comparative Analysis of Human Papillomavirus Type 18 Isolated from a Nasopharyngeal Carcinoma from South Africa

**DOI:** 10.1128/MRA.00630-21

**Published:** 2021-09-30

**Authors:** Yuri Munsamy, Riaz Y. Seedat, Tumelo R. Sekee, Phillip A. Bester, Danelle van Jaarsveldt, Felicity J. Burt

**Affiliations:** a Division of Virology, Faculty of Health Sciences, University of the Free State, Bloemfontein, South Africa; b Department of Otorhinolaryngology, Faculty of Health Sciences, University of the Free State, Bloemfontein, South Africa; c Department of Otorhinolaryngology, Universitas Academic Hospital, Bloemfontein, South Africa; d Division of Virology, National Health Laboratory Service, Universitas, Bloemfontein, South Africa; DOE Joint Genome Institute

## Abstract

We report the complete genome sequence of human papillomavirus type 18 isolated from a nasopharyngeal carcinoma in South Africa.

## ANNOUNCEMENT

Human papillomaviruses (HPVs) belong to the family *Papillomaviridae* and have small, double-stranded, circular DNA genomes of approximately 8 kb that contain 8 or 9 open reading frames (ORFs) (1). Within the genus *Alphapapillomavirus*, HPV18 is the second most frequently identified type in cervical carcinomas and HPV-associated head and neck squamous cell carcinomas (HNSCCs) worldwide (2–5). It has been suggested that HPV may play a role in non-Epstein-Barr virus-related nasopharyngeal carcinoma (6). There are limited data regarding the genomic diversity of HPV isolates from HNSCCs compared to isolates from cervical cancers. Whole-genome data are available for isolates from cervical cancers but not from HNSCCs. Here, the whole-genome sequence of an HPV18 isolate from a nasopharyngeal carcinoma, obtained using next-generation sequencing (NGS), is described. Ethics approval for conducting this study was obtained from the Ethics Committee of the Faculty of Health Sciences, University of the Free State (ECUFS NR 137/2013D).

Biopsy tissue was collected from a patient with nonkeratinizing squamous cell carcinoma of the nasopharynx at Universitas Academic Hospital (Bloemfontein, South Africa) and designated VBD17/15. DNA was extracted from fresh biopsy tissue using the QIAamp DNA minikit (Qiagen, USA) according to the manufacturer’s instructions. The full-length genome sequence was amplified in two overlapping fragments (genes E1 to L1; genes L1 to E1), with primers designed in-house specifically for HPV18 (F1, 5′-GGAGATTGGAGACCAATAGTG-3′; R1, 5′-CATATTGCCCAGGTACAGGAG-3′; F2, 5′-ATTCTCCCTCTCCAAGTGGC-3′; R2, 5′-CATCTAACATGGCCACCTTAG-3′), using the Phusion HotStart DNA polymerase-mediated PCR amplification kit (Finnzymes, Finland) according to the manufacturer’s instructions.

The genomic DNA library was prepared using the Nextera XT DNA library preparation kit (Illumina, USA), followed by size selection using AMPure XP beads (Beckman Coulter, USA). The multiplexed libraries were analyzed on a MiSeq sequencer (Illumina) with the MiSeq reagent kit v3 (300 cycles) (Illumina). The length range was 167 bp, while the mode length was 201 bp, with 135,173 sequences and an average coverage of 400×. PRINSEQ was used to trim and filter reads based on length and quality scores (≥QC30) (7). The contigs assembled *de novo* (SPAdes v3.7.1) from the respective overlapping amplicons were assembled using cap3 after removing the primer-binding sites from both these contigs (8). The original reads were mapped to the resulting consensus genome sequence to look for coverage of at least 100× and to determine if any ambiguities were present (9). Ample coverage and the absence of ambiguities were confirmed. The annotate and predict function in Geneious Prime v7.0 (Biomatters Ltd., New Zealand) was used for annotation of the consensus genome using the genome submitted under GenBank accession number NC_001357 as the reference. All tools were run with default parameters unless otherwise specified.

The complete genome sequence of VBD17/15 was shown to share 99.8% nucleotide identity to the HPV18 reference genome (GenBank accession number NC_001357). VBD17/15 was 7,857 kb in length with 9 fully identified genes and a GC content of 40.42%. The HPV18 reference isolate (NC_001357) and VBD17/15 were compared, and 14 genetic variations were identified (0.19% of genome). A nucleotide substitution in VBD17/15, T4315A, was completely novel to this South African isolate.

The findings of this study contribute knowledge regarding HPV18 isolates associated with HNSCCs (10, 11) and lay the groundwork for future research into HPV-associated HNSCC genomic characterization in South Africa.

### Data availability.

The sequence reads were submitted to the European Nucleotide Archive under the following accession numbers: ERX6128830, ERX6128831, ERR6501609, and ERR6501610. This complete genome sequence has been deposited in GenBank under the accession number MW836821.
